# Virome Analysis for Identification of a Novel Porcine Sapelovirus Isolated in Western China

**DOI:** 10.1128/spectrum.01801-22

**Published:** 2022-08-08

**Authors:** Jianing Chen, Xuepeng Suo, Liyan Cao, Cong Yuan, Lei Shi, Yueyue Duan, Haixue Zheng, Qi Wang

**Affiliations:** a Institute of Urban Agriculture, Chinese Academy of Agricultural Sciences, Chengdu, China; b State Key Laboratory of Veterinary Etiological Biology, National Foot and Mouth Diseases Reference Laboratory, Lanzhou Veterinary Research Institute, Chinese Academy of Agricultural Sciences, Lanzhou, China; c Chengdu National Agricultural Science and Technology Center, Chengdu, China; Changchun Veterinary Research Institute

**Keywords:** biological characterization, diarrhea, next-generation sequencing, porcine sapelovirus

## Abstract

Diarrhea is one of the most important problems associated with the production of piglets, which have a wide range of possible pathogens. This study identified a strain of porcine sapelovirus (PSV) by using next-generation sequencing (NGS) technologies as the pathogen among fecal samples in a pig herd. Phylogenetic analysis showed that the PSV isolates shared a unique polyprotein and clustered with Chinese isolates identified before 2013. The PSV strain was then isolated and named GS01. The *in vitro* and *in vivo* biological characteristics of this virus were then described. Our pathogenicity investigation showed that GS01 could cause an inflammatory reaction and induce serious diarrhea in neonatal piglets. To our knowledge, this is the first isolation and characterization of PSV in western China. Our results demonstrate that the PSV GS01 strain is destructive to neonatal piglets and might show an expanded role for sapeloviruses.

**IMPORTANCE** Porcine sapelovirus (PSV) infection leads to severe polioencephalomyelitis with high morbidity and mortality, resulting in significant economic losses. In previous studies, PSV infections were always subclinical or only involved a series of mild symptoms, including spinal cord damage, inappetence, diarrhea, and breathless. However, in our study, we isolated a novel PSV by virome analysis. We also determined the biological characteristics of this virus *in vitro* and *in vivo*. Our study showed that this novel PSV could cause an inflammatory response and induce serious diarrhea in neonatal piglets. To our knowledge, this is the first isolation and characterization of PSV in western China. These findings highlight the importance of prevention for the potential threats of PSV.

## INTRODUCTION

Diarrhea is a common but serious gastrointestinal disease in pigs, especially for suckling piglets. The main clinical symptoms include diarrhea, dehydration, and poor growth, even upon recovery. The morbidity and mortality vary among different causative agents. To date, with the development of drugs and facilities, bacteria and parasites are hardly the main factors contributing to diarrhea. On the contrary, more and more viruses are identified to be associated with enteric diseases. The most destructive enteric viruses, including porcine epidemic diarrhea virus (PEDV), swine acute diarrhea syndrome coronavirus (SADS-CoV), transmissible gastroenteritisvirus (TGEV), porcine rotavirus (PRoV), and porcine deltacoronavirus (PDCoV), can result in a death rate from 40% to 100% (1–4). Moreover, several other viruses, including porcine sapelovirus (PSV), porcine enteroviruses (PEVs), porcine teschoviruses (PTVs), porcine astrovirus (PAstV), porcine bocavirus (PBoV), porcine Norwalk virus (PNoV), porcine Kobuvirus (PKV), and many others, may lead to mild diarrhea and associated symptoms (5–12). However, multiple pathogens may combine to form a coinfection that is difficult to diagnose. The precise examination of the causative agent of porcine diarrhea is difficult to determine.

The metagenomics approach, based on next-generation sequencing (NGS) technology, is an effective strategy for the detection of all potential pathogens in a sample. It was used in various independent studies for the detection of viruses in pig samples and many others (13–16). The recent outbreak of severe acute respiratory syndrome coronavirus 2 was also first revealed by NGS (17). This method may further serve to identify novel infections and characterize the animal virome in both healthy and diseased states.

In this study, diarrhea samples containing unknown pathogens were collected from a pig farm in Gansu Province, China. After the failure of target detection of PEDV, TGEV, PDCoV, SADS-CoV, and PRoV, which was performed by reverse transcription-PCR (RT-PCR), the samples were then subjected to NGS. PSV was identified as the causative agent and successfully isolated. The molecular and biological characterization of this PSV isolate are systematically described here.

## RESULTS

### NGS revealed that PSV was involved in an unidentified diarrhea.

**FIG 1:**
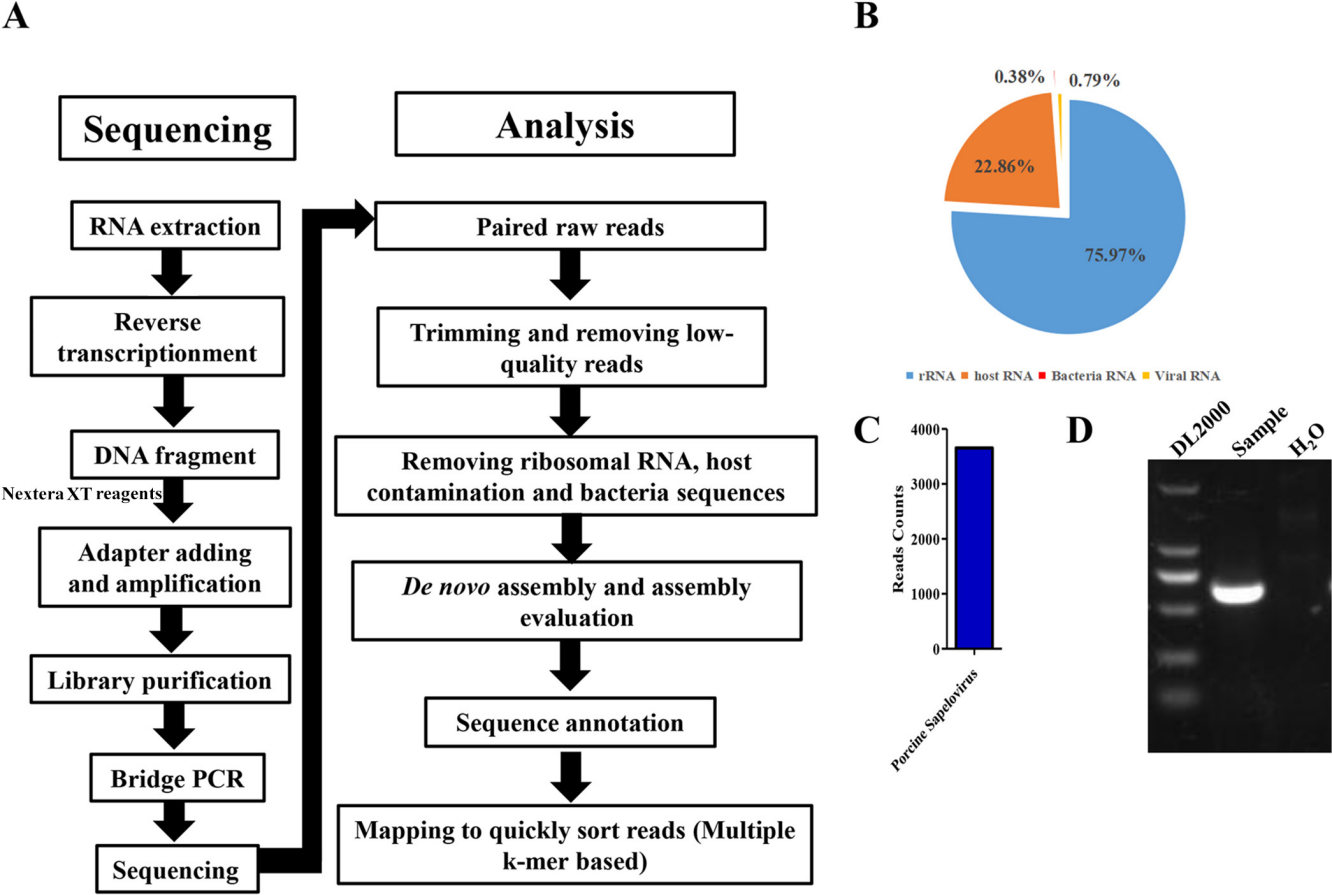
Next-generation sequencing identified PSV in diarrhea samples. (A) Workflow of next-generation sequencing for the virome. (B) Statistical results for different RNAs obtained by NGS. (C) The viral annotation showed porcine sapelovirus A (PSV) was the only identified virus. (D) RT-PCR results confirmed that PSV genome RNA existed in diarrhea samples.

In March 2021, diarrhea of unknown pathogens broke out in a pig farm in Gansu Province, China. Although no obvious clinical symptoms were observed in sows, suckling piglets ≤21 days old displayed serious watery diarrhea and dehydration. The death rate of suckling piglets was about 10%. The recovered pigs presented with growth retardation. None of the viruses PEDV, TGEV, PDCoV, SADS-CoV, PCV2, PCV3, PCV4, or rotavirus were detected in samples via PCR analysis. Therefore, collected samples were prepared for NGS, analyzed, and searched against the NCBI NT database to figure out the potentially associated pathogens (Fig. 1A). The results demonstrated that the NGS obtained 6,032,598 clean reads (Fig. 1B). With the removal of rRNA, host contamination, and bacterial sequences, the remaining 3,654 reads all mapped to the genome of PSV A (Fig. 1C). Further validation also proved the existence of PSV among the diarrhea samples by RT-PCR (Fig. 1D). These data above suggested that sapelovirus A might correlate with the outbreak of diarrhea among the pig herd.

### Isolation and identification of PSV strain GS01.

**FIG 2:**
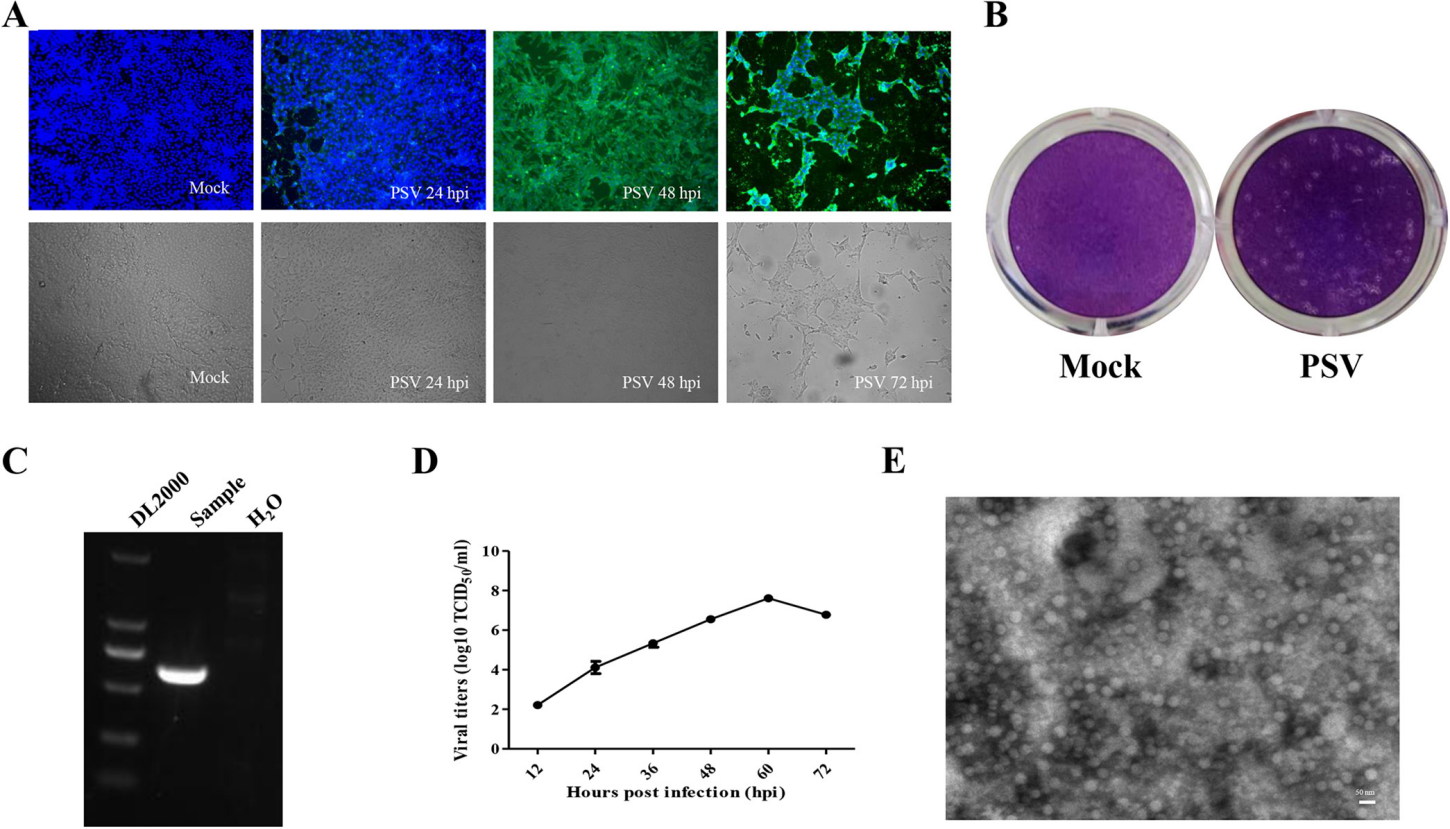
Isolation and characterization of PSV/GS01/China/2021. (A) LLC-PK1 cells grown to 90% confluence were inoculated with filtered diarrhea samples. The cells displayed obvious CPE from 48 hpi. IFA results showed there were positive signals distributed among cells. (B) RT-PCR detection of PSV RNA in the supernatant. (C) PSV GS01 strain was purified by plaque assay. (D) LLC-PK1 cells were infected with PSV GS01 at a multiplicity of infection of 0.01. The supertanant was harvested at 12, 24, 36, 48, 60, and 72 hpi to determine the growth kinetics curve for PSV GS01. (E) Electron microscopy of purified PSV GS01 virions.

PK1 cells grown to 85% confluence were inoculated with filtered samples and replaced with fresh medium at 1 h postinfection (hpi). The cells displayed visible cytopathic effect (CPE) from 36 hpi. CPE included shrinking, rounding, and detachment of all cells from 48 hpi. An immunofluorescence assay (IFA) confirmed the existence of PSV-VP1 in inoculated cells (Fig. 2A). The supertanant was then collected and blindly passaged in PK1 cells. After five passages, CPE was still displayed by inoculated cells. Therefore, the viral stock was purified by plaque assay (Fig. 2B). The purified virus was subjected to another amplification to obtain more purified viruses. RNA extracted from the purified virus was positive for PSV (Fig. 2C). The growth characteristics of PSV were described by the growth kinetics curve. The infection of PSV was confirmed by IFA analysis. The results showed that the viral titers peaked at 60 hpi with about 10^7.5^ 50% tissue culture infective doses (TCID_50_), suggesting the PSV strain could efficiently replicate in PK1 cells (Fig. 2D). To determine the morphological characteristics of PSV, virions were negatively stained and examined by transmission electron microscopy, which showed that the viral particles were nonenveloped and approximately 30 to 50 nm in diameter (Fig. 2E). All these data confirmed that the PSV GS01 strain was successfully isolated.

### Phylogenetic analyses of PSV GS01.

For further analysis of the genomic characteristics of PSV GS01, its complete genome sequence was assembled by NGS and later submitted to GenBank (accession number OM328111). The full genome of PSV GS01 was 7,530 bp in length, contained a 5′ untranslated region (UTR) of 474 bp, a polyprotein gene of 6,996 bp, and a 3′-UTR of 60 bp. Compared with other PSV strains, GS01 exhibited shorter 3′-UTR sequences. The length of the 5′-UTR was 489 to 491 bp, which was 80 to 120 bp shorter than the usual 3′-UTR for other strains. The phylogenetic tree based on the sequence of the polyprotein gene was then constructed with reference strains from China and other countries.

**FIG 3:**
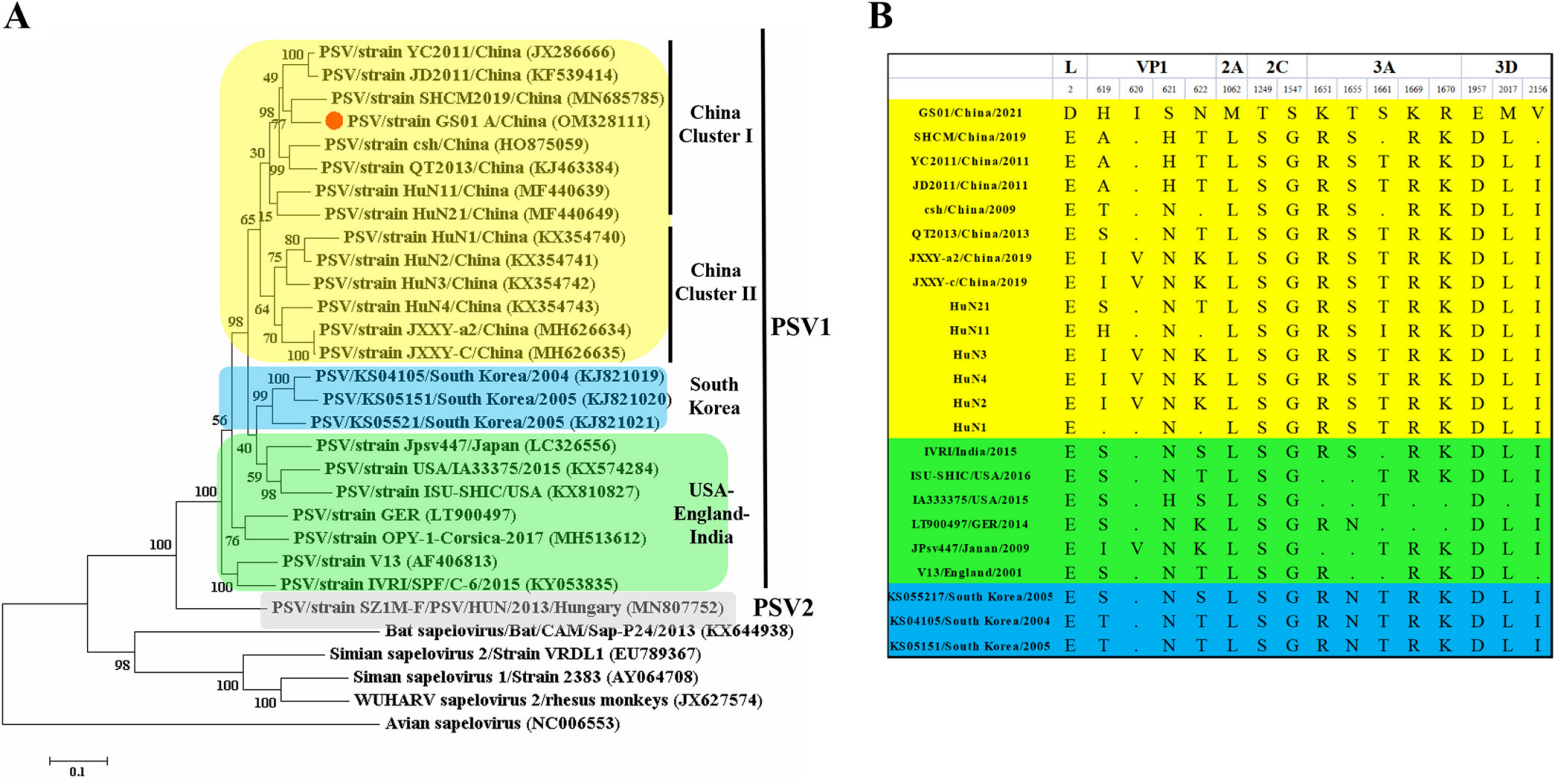
Phylogenetic analysis of the PSV GS01 strain, comparing the nucleotide sequences of the polyprotein coding regions of PSV strains, conducted with MEGA7.0 software. (A) The phylogenetic tree was constructed using the maximum-likelihood method with 1,000 bootstrap replicates. The PSV strain isolated in this study is indicated by a red dot. All Chinese PSV isolates are indicated by a yellow box and South Korean PSV isolates are indicated by a blue box, while England-USA-India PSV isolates are indicated by a green box. All other members of the genus *Sapelovirus* are either unshaded or are indicated by a gray box. (B) Differences in the amino acid sequences for the polyproteins of the PSV GS01 strain and reference strains. The yellow bar indicates the Chinese strain. The green bar indicates the England-USA-India strains, while the blue bar indicates the South Korean strains.

The results showed that there existed two genotypes of PSV, PSV1 and PSV2. PSV1 contained most of the identified PSV strains, while PSV2 only contained PSV strains isolated from Hungary (18). PSV1 strains formed three distinct clusters, including the Chinese cluster, the South Korea cluster, and the USA-England-India cluster. The Chinese cluster contained two subclusters. The strains identified mainly before 2013 formed subcluster I, while strains identified in Hunan and Jiangxi Provinces formed subcluster II. Most of subcluster II strains were identified after 2014. The PSV GS01 strain was classified in subcluster I and was closely related PSV SHCM strain. Only these two strains within subcluster I were identified after 2013 (Fig. 3A). The deduced amino acid sequence of the polyprotein was also compared with that of reference strains. The results showed that there existed one mutant cluster within VP1 of GS01 strain. The mutant cluster included four adjacent amino acids located at positions 619 to 622 which were not observed in other strains. There were also 5 unique amino acid substitutions, including an E-to-D change at position 2 (E2D), L1063M, S1249T, G1547S, and D1957E; these were only identified in the GS01 strain. The amino acid substitutions R1651K, S1655T, N1655T, T1661S, R1669 K, K1670 R, L2017M, and I2156V were observed in GS01 and other very limited strains. Interestingly, the substitutions R1651K, S1655T, N1655T R1669K, K1670R, and L2017M were only present in GS01 and strains within the USA-England-India cluster (Fig. 3B). As these represent strains of subcluster II, the known pathogenic strain PSV-JXXY-a2 had several mutant clusters that were not identified in GS01 strain. We compared nucleotides and amino acid identities for GS01 between different strains of porcine sapelovirus A (Table 1). Moreover, Recombination Detection Program version 4 (PRD4) was used to detect the recombination events in the genome of GS01. The results showed that no recombination signal was detected within the GS01 genome (data not shown). The results above further indicated that PSV GS01 was an emerging strain in western China.

**TABLE 1:** Comparison of the nucleotides and amino acid identities between GS01 and different strains of porcine sapelovirus A

Strain	Sequence homology (%) for:	
	Nucleotides	Amino acids
YC2011	90.0	98.0
JD2011	89.8	97.9
SHCM2019	89.7	98.2
CSH	89.9	97.7
OT2013	89.8	98.0
HuN11	88.8	97.5
HuN21	88.0	96.8
HuN1	88.9	96.7
HuN2	87.8	94.6
HuN3	87.5	95.0
HuN4	87.5	94.9
JXXY-a2	87.6	94.7
JXXY-C	87.6	94.6
KS04105	87.1	97.1
KS05151	87.5	97.3
KS05521	86.6	95.7
Jpsv447	85.2	94.0
IA33375	86.0	95.7
ISU-SHIC	84.9	93.9
GER	84.9	93.3
OPY-1-Corsica-2017	84.6	93.5
V13	84.0	93.2
IVRI/SPF/C-6/2015	84.2	93.5
SZ1M-F/PSV/Hun/2013	76.6	84.7

### PSV GS01 is pathogenic to neonatal piglets.

**FIG 4:**
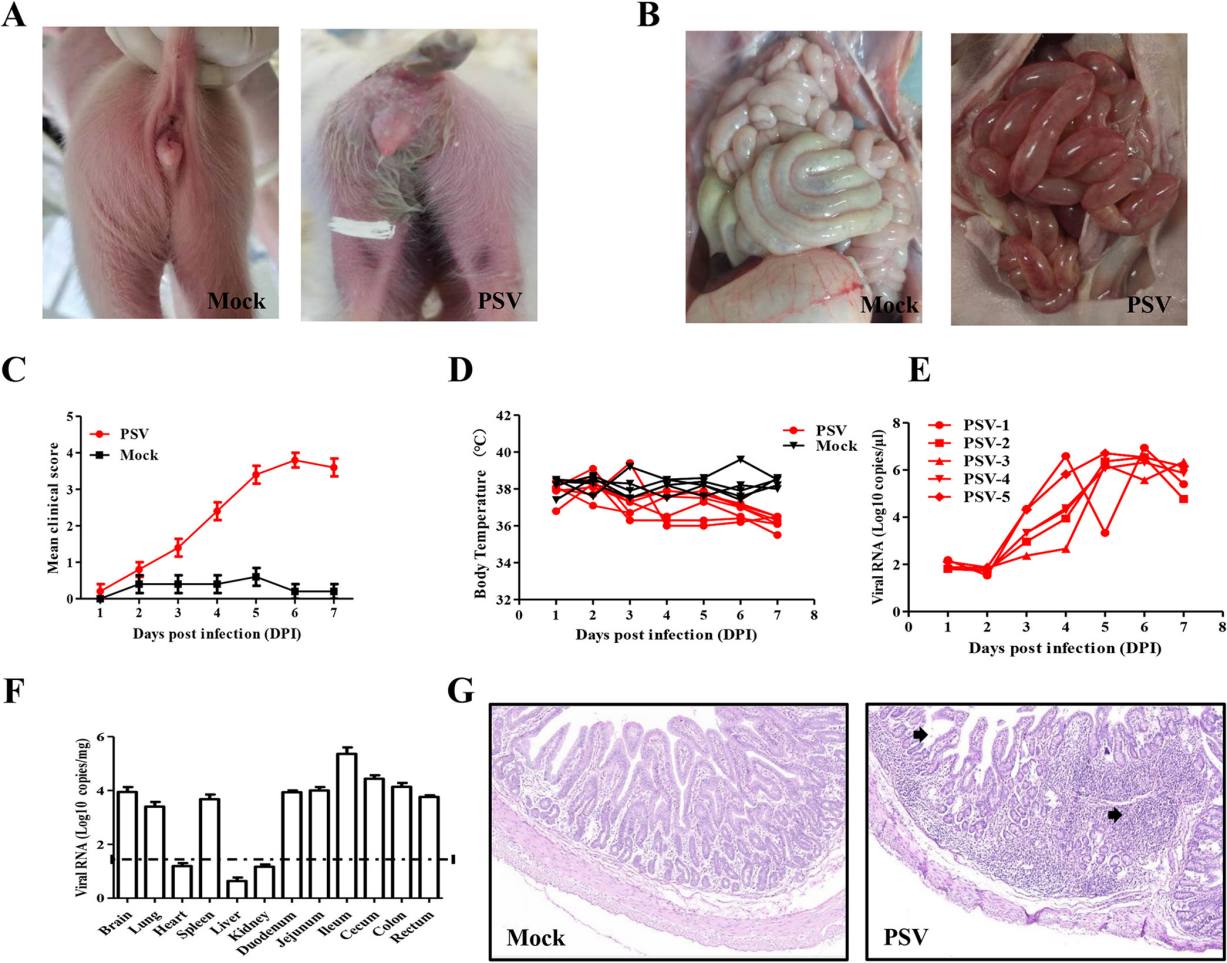
Pathogenic analysis of PSV/GS01/China/2021-infected suckling piglets. Twelve-day-old piglets were orally inoculated with PSV GS01 (10^6^ TCID_50_/pig) or an equal volume of MEM 199. (A to D) Clinical symptoms (A and B), clinical scores (C), and body temperature (D) were observed daily. (E) Fecal swabs were also collected for the detection of shedding virus. (F) At 7 dpi, all piglets were euthanized, and different organs were collected for viral load determinations. (G) Small intestines were also prepared for histopathological analysis.

To determine whether the isolated PSV GS01 strain was the causative agent of diarrhea in suckling piglets, five 12-day-old neonate piglets negative for PSV, PEDV, TGEV, PDCoV, SADS-CoV, and PRoV were orally inoculated with 10^6^ TCID_50_ of PSV GS01 strain. Another five piglets were inoculated with an equal volume of minimal essential medium 199 (MEM 199) as the control. All piglets were observed daily for clinical scores and body temperature. The clinical symptoms were evaluated using 5 grades (0 = healthy, 1 = soft but formed feces, 2 = mild diarrhea, 3 = watery diarrhea, 4 = paralyzation, 5 = moribund or dead). The fecal swabs were also collected daily to determine virus shedding. The results showed that no significant clinical symptoms were observed among the challenged piglets compared to control piglets during the first 3 days. The clinical scores demonstrated that the physical situations of piglets became polarized after 3 days postinfection (dpi) (Fig. 4C). At 4 dpi, all inoculated piglets developed severe diarrhea but kept suckling (Fig. 4A). At 6 dpi, three of the inoculated piglets failed to stand and remained lying all day. The decrease of body temperature was also observed following 6 dpi (Fig. 4D). Therefore, all piglets were euthanasized at 7 dpi. The small intestines of inoculated piglets became transparent and filled with air while thick and full of content in challenged piglets. Hemorrhage was observed among the mesentery and small intestine (Fig. 4B). In contrast, other tissues did not exhibit visible pathological changes. The real-time PCR analysis demonstrated that the viral shedding initiated at 1 dpi, increased from 3 dpi, and peaked at 5 and 6 dpi (Fig. 4E). Moreover, PSV was found to be widely distributed among different tissues. High numbers of RNA copies of PSV were detected in brain, lung, spleen, and whole intestine but not in the heart, liver, or kidney (Fig. 4F). In addition, histopathological results showed that lots of the inflammatory cells aggregated among the intestine and villi in the small intestine were significantly atrophic, suggesting that the inflammatory reaction was induced by PSV infection (Fig. 4G). All these results demonstrated that PSV infection resulted in the diarrhea of suckling piglets.

## DISCUSSION

In this study, NGS methods were used to detect unknown pathogens of porcine diarrhea samples. PSV was the only identified virus among the samples. The PSV GS01 strain was then successfully isolated. Phylogenetic analysis demonstrated that the isolate could be classified into a Chinese subcluster that contained PSV strains rarely identified since 2013. A new mutant cluster was identified within the VP1 of GS01 strain. The pathogenic examination further showed this virus could induce serious diarrhea threatening to suckling piglets.

PSV is a nonenveloped, positive-sense, single-stranded RNA virus. PSV belongs to the genus *Sapelovirus* in the family *Picornaviridae*, according to the taxonomic criteria system of the International Committee on Taxonomy of Viruses (19). The virus was initially identified in the United Kingdom in 1958 and later found to be circulating worldwide (20–24). Pigs of all ages can be infected by PSV. In infected piglets, the virus has been detected among different tissues except for the liver, kidney, and heart (25, 26). Therefore, it is associated with various kinds of disorders, including respiratory distress, reproductive failure, polioencephalomyelitis, and most importantly, diarrhea (5, 27, 28). However, the symptoms induced by PSV infection are usually subclinical manifestations and may often be ignored. The coinfection of PSV and other pathogens may result in more serious consequences to piglets.

PSV was first isolated in China in 2009 (23). More and more information of its circulation in southern China has been provided since then. PSV was frequently detected in fecal samples in Hunan, China (7, 29). In 2019, two different PSV strains, JXXY-a2 and JXXY-c, associated with only diarrhea, were isolated in Jiangxi Province (30). The subsequent epidemiological investigation showed that PSV was prevalent in southern China. The positive rate of PSV was about 45% in diarrhea samples and 21% in asymptomatic samples (7, 25). Phylogenetic analysis showed the isolates in Hunan and Jiangxi varied from previous Chinese strains (cluster I) and formed another cluster (cluster II). These strains are characterized by three mutant clusters among VP1. Recently, several PSV strains were isolated in Heilongjiang, China. Serum investigation has also suggested PSV was prevalent in northern China (31). Although PSV, especially cluster II strains, are emerging in southern China, their pathogenicity to piglets has not been fully explored.

There was little existing information about the emergence of PSV in western China. In this study, PSV was isolated from piglets suffering from diarrhea in Gansu, China. This is the first report about the emergence of PSV in western China. The identified PSV GS01 strain was classified as cluster I, which has been rarely detected since 2013. The pathogenicity of the cluster I strain also had not been tested except for one case (26). In this study, the single infection of PSV GS01 strain resulted in severe watery diarrhea in suckling piglets. The villi in the small intestine were significantly atrophic. Moreover, the inflammatory reaction was also induced by PSV infection. Although virus was detected in the lungs and other organs, no visible pathological changes were observed in these tissues. The outbreak of GS01 strain caused a death rate of approximate 10% in a pig farm without the coinfection of other viruses. The strain also displayed high pathogenicity to suckling piglets during the animal experiment. These results suggested that, compared to other strains, the PSV GS01 strain has a higher pathogenicity to suckling piglets.

To date, PSV has been poorly studied. PSV infection is often ignored due to its subclinical manifestations. Both the epidemiology and the pathogenicity are not well characterized. To our knowledge, PSV GS01 strain is the first strain identified in western China. The characterization of PSV GS01 strain suggests that PSVs of different clusters are emerging in China, and some strains are highly pathogenic to suckling piglets. With increasingly frequent isolation of PSV since 2019, a zoonosis of PSV seems to have originated in China (25, 26, 30, 31). Therefore, more attention should be paid to the emergence of PSV.

## MATERIALS AND METHODS

### Sample collection.

In March 2021, diarrhea broke out in a pig farm in Gansu Province, China. In this farm, suckling piglets ≤21 days old displayed watery diarrhea, vomiting, and dehydration. The gross lesions were mainly found in the intestine and included edema and vascular engorgement among the mesentery; thickening of the bowel wall was not observed in other tissues. About 10% of piglets died 5 to 7 days after the beginning of diarrhea. The surviving pigs presented with growth retardation. No obvious clinical symptoms were observed in sows. Watery feces were collected from pigs at 5 days postdiarrhea and were sent for detection. None of the viruses PEDV, TGEV, PDCoV, SADS-CoV, PCV2, PCV3, PCV4, or rotavirus were detected by RT-PCR. The primers used for detection are listed in Table 2. The collected samples were centrifuged at 2,000 rpm for 3 min, and clarified supernatant was then filtered with a 0.45-μm filter. The supertanants were mixed and sent for next-generation sequencing.

**TABLE 2:** Primers for virus detection

Virus	Forward primer	Reverse primer
PEDV	TCAACAGCTTCCCAGCGTAG	GCTGCTACGCTATTTTCGCC
TGEV	ATGGTAAAGTGCCAGGCGAA	ATTGGCAACGAGGTCAGTGT
PDCoV	CCTCATGTTGCCAAACGCAA	CCCTTGGGTAAAGTCCGCTT
SADS-CoV	ACGGATTCAGGGTGTGCATT	GTTGAGCACGAGGTGTCTGA
Rotavirus	GCGGATTTGGTGCAACCTTT	ACGTTAATTGGGTCAGCCGT
PCV-2	AAGGGCTGGGTTATGGTATG	CGCTGGAGAAGGAAAAATGG
PCV-3	GGCCTTGGTGGGATGGTTAT	TTTCTCCGGCTCAGCAAACA
PCV-4	GGGGGTTCCATTGAGTTCGT	CCTCTTGGAGCGTTGGACAT
PSV	GATGTGGCGCATGCTCTT	TGCTGCCTCCTGTGTTGTTAT

### Next-generation sequencing.

For NGS, total RNA was extracted from samples with TRIzol (Invitrogen, USA) and reverse transcribed used random hexamers, following DNase treatment and second-strand synthesis. Nextera XT reagents (Illumina) were used for library preparation. Briefly, DNA was first tagged and fragmented by using the Nextera XT transposon, followed by the addition of adapter sequences to the ends. The tagmented DNA was then amplified via a limited cycle PCR program. AMPure XP beads were used to remove the very short library fragments to purify the library DNA. The library was finally normalized and loaded for sequencing. Sequencing was performed with the NovaSeq 6000 system (Illumina) by Shanghai Tanpu Biotechnology Co., Ltd. (Shanghai, China). Bioinformatics analysis of the data was completed using the pipeline described in Fig. 1A. Briefly, after removal of low-quality reads, rRNA, host RNA, and bacteria RNA, MetaSPAdes software was used to *de novo* assemble the remaining reads. The final scaffolds were subjected to a MegaBLAST homology search against the NCBI NT database.

### Virus isolation and purification.

LLC-PK1 cells were purchased from the China Center for Type Culture Collection and maintained in our laboratory. The cells were cultured in MEM 199 (Gibco, USA) supplemented with 0.1 U/mL bovine insulin and 10% fetal bovine serum (Gibco, USA). For virus isolation, LLC-PK1 cells were inoculated with the filtered samples and observed daily for 3 days to monitor the development of CPE. At 3 dpi, cells were subject to three freeze-thawing cycles. The supertanant was then collected. After five passages in LLC-PK1 cells with CPE appearance, the viral stock was used for purification. Briefly, confluent monolayers of PK1 cells were incubated with viral stock for 1 h at 37°C on a rocker. After washing with phosphate-buffered saline (PBS), the cells were overlaid with 0.75% low-melting-point agarose (Sigma, Germany) in MEM 199 containing 5% FBS and incubated at 37°C for 72 h. To obtain purified virus, the agarose within a single plaque was collected and used for viral amplification. The primers used for PSV detection are listed in Table 2.

### Electron microscopy.

The culture medium of PSV-infected cells was collected and centrifuged at 12,000 rpm for 5 min to remove cell debris. The supertanant was then concentrated at 10,0000 x g for 2 h, at 4°C. The pellet was resuspend in PBS and purified by sucrose density gradient centrifugation. The sucrose cushion was 20% and 40%. PSV particles were below the 40% sucrose cushions. The pellet was resuspended in PBS and spotted onto Formvar-coated grids for 10 min followed by negative staining with phosphotungstic acid for another 10 min at room temperature. The grids were finally subjected to observation with a Hitachi electron microscope (HT7700) at 80 kV.

### Immunofluorescence assay.

LLC-PK1 cells were seeded in 12-well plates and infected with filtered samples at 37°C. At different time points, cells were fixed with 4% paraformaldehyde and permeabilized with 0.5% Triton X-100 at room temperature. The cells were then blocked with 5% skim milk and incubated with rabbit anti-PSV-VP1 polyclonal antibody (1:1,000 dilution), followed by Alexa Fluor 488 goat anti-rabbit IgG antibody (1:1,000 dilution; Thermo Fisher, USA) for 1 h. The cell nuclei were stained with 4′,6-diamidino-2-phenylindole (Beyotime, China) and examined with a fluorescence microscope (TE2000U; Nikon) with a video documentation system.

### Viral titration.

PSV was made into serial 10-fold dilutions between 10^−1^ and 10^−10^ and inoculated in LLC-PK1 cells. After incubation at 37°C for 1 h, the cells were washed with PBS three times and incubated for another 48 h. Then, the cells were fixed and subjected to an immunofluorescence assay. Finally, the results were calculated based on the Reed-Muench method.

### Phylogenetic analysis.

The sequences of PSV GS01 (GenBank accession OM328111) and reference strains were aligned with the Molecular Evolutionary Genetics Analysis (MEGA) 7.0 tool (multiple alignment using ClustalW) (32). The phylogenetic trees were constructed using the maximum-likelihood method with 1,000 bootstrap replicates. The information for selected reference strains is listed in Table 3.

**TABLE 3:** Reference strains of sapelovirus used in this study

GenBank accession no.	Strain	Date	Country
JX286666	YC2011	2011	China
KF539414	JD2011	2011	China
MN685785	SHCM2019	2019	China
HQ875059	CSH	2009	China
KJ463384	OT2013	2013	China
MF440639	HuN11	2014	China
MF440649	HuN21	2014	China
KX354740	HuN1	2015	China
KX354741	HuN2	2015	China
KX354742	HuN3	2015	China
KX354743	HuN4	2015	China
MH626634	JXXY-a2	2017	China
MH626635	JXXY-C	2017	China
KJ821019	KS04105	2004	South Korea
KJ821020	KS05151	2005	South Korea
KJ821021	KS05521	2005	South Korea
LC326556	Jpsv447	2009	Japan
KX574284	IA33375	2015	USA
KX810827	ISU-SHIC	2017	USA
LT900497	GER	2017	Germany
MH513612	OPY-1-Corsica-2017	2017	India
AF406813	V13	2002	Germany
KY053835	IVRI/SPF/C-6/2015	2015	India
MN807753	SZ1M-F/PSV/Hun/2013	2013	Hungary
KX644938	CAM/Sap-P24/2013	2013	Cameroon
EU789367	Simian sapelovirus 2 strainVRDL1	2008	USA
AY064708	Simian sapelovirus 1 strain 2383	2003	USA
JX627574	WUHARV sapelovirus 2	2012	USA
NC006553	Avian sapelovirus	2004	USA

### Animal experiments.

For pathogenic examination, 10 pigs of 12 days old were purchased from a farm negative for PSV, PDCoV, PEDV, TGEV, and SADS-CoV. Five pigs were orally inoculated with PSV GS01 (10^6^ TCID_50_/pig), while five pigs belonging to the control group were orally inoculated with an equal volume of MEM 199. Clinical symptoms were observed daily. At 7 dpi, all pigs were euthanized and different organs were collected for viral load determinations. Fecal swabs and tissues were also collected for viral load determinations. The small intestine was analyzed for microscopic lesions. All animal experiments were performed in accordance with the Experimental Animal Ethics Committee of Lanzhou Veterinary Research Institute.

### Real-time PCR analysis.

For the quantitation of viral shedding and viral loading among different tissues, the real-time PCR analysis was performed in an Applied Biosystems QuantStudio 5 system with Premix *Ex Taq* (TaKaRa, Japan). Briefly, the reaction mixtures were incubated at 95°C for 30 s, followed by 40 cycles at 95°C for 5 s and 60°C for 30 s. The sequences of primers and probe were as follows: PSV-F (5′-GGATTACACCAATGCACAG-3′), PSV-R (5′-CCAGTATGTAAAGGCTCT-3′), probe (5′–6-carboxyfluorescein–TCCTTGCTGTGGTCCTCTGC–6-carboxytetramethylrhodamine–3′).

### Data availability.

The complete genome data used in this study are available in the NCBI Sequence Read Archive (SRA) database under BioProject number PRJNA813904.
