# Supplementary material for: Virome Analysis for Identification of a Novel Porcine Sapelovirus Isolated in Western China
Source: Microbiol Spectr. 2022 Aug 8;10(4):e01801-22. doi: 10.1128/spectrum.01801-22 (PMC9430179; doi:10.1128/spectrum.01801-22)
Supplement: Supplemental file 1 — Supplemental material. Download spectrum.01801-22-s0001.pdf, PDF file, 0.3 MB [file spectrum.01801-22-s0001.pdf]

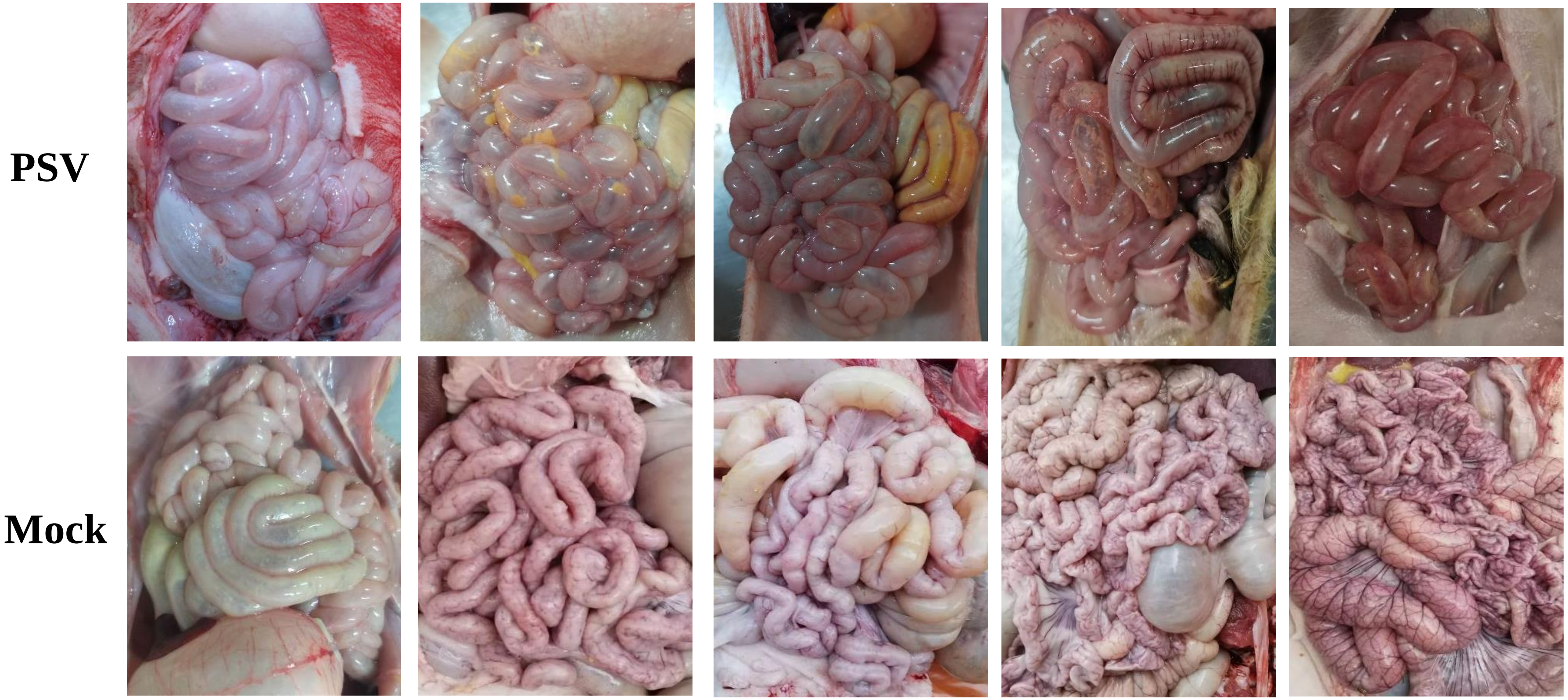

Supplementary Fig. 1

The gross lesions to the intestine of PSV/GS01/China/2021 infected piglets. At 7 dpi, all of the piglets were euthanatized. All challenged piglets had serious hemorrhage and congestion of the small intestine. The wall of the intestine turned to thinning in challenged piglets compared to the control.
